# Supplementary material for: Metabolic Stability of New Mito-Protective Short-Chain Naphthoquinones
Source: Pharmaceuticals (Basel). 2020 Feb 12;13(2):29. doi: 10.3390/ph13020029 (PMC7169385; doi:10.3390/ph13020029)
Supplement: Supplementary file 1 [file pharmaceuticals-13-00029-s001.pdf]

Supplementary Materials

Metabolic Stability of New Mito-Protective Short-Chain Naphthoquinones

Zikai Feng [1,2,3], Jason A. Smith [2], Nuri Gueven [3], and Joselito P. Quirino [1,2,*]

1 Australian Centre for Research on Separation Sciences (ACROSS), School of Natural Sciences-Chemistry, College of Science and Engineering, University of Tasmania, Hobart, TAS 7000, Australia
2 School of Natural Sciences-Chemistry, College of Science and Engineering, University of Tasmania, Hobart, TAS 7000, Australia
3 Division of Pharmacy, School of Medicine, College of Health and Medicine, University of Tasmania, Hobart, TAS 7000, Australia
* Correspondence: jquirino@utas.edu.au

Table S1. Analytical figures of merit for the RP-LC of 16 new short-chain quinones (SCQs) and the clinical used benzoquinone idebenone for the metabolic stability study.

Compound	ID	RT	Equation	R^2	***c*** (μM) found at ***t*** = 0	Recovered %	RSD %
**1**	UTAS#81	3.75	Y = 0.5063*X - 0.1593	0.996	11.5 ± 0.7	115.1 ± 7.0	6.1
**2**	UTAS#80	3.75	Y = 0.7420*X - 0.3726	0.999	10.5 ± 0.4	105.2 ± 3.9	3.7
**3**	UTAS#62	4.18	Y = 1.0500*X - 0.1085	1.000	10.9 ± 0.9	109.4 ± 8.8	8
**4**	UTAS#78	4.18	Y = 0.2149*X - 0.1450	0.995	11.0 ± 0.4	110.5 ± 4.3	3.9
**5**	UTAS#37	5.36	Y = 1.0230*X - 0.1533	0.999	11.1 ± 0.1	110.6 ± 1.3	1.2
**6**	UTAS#72	6.62	Y = 0.8226*X - 0.1267	0.999	11.4 ± 0.3	114.2 ± 3.2	2.8
**7**	UTAS#74	4.30	Y = 0.6179*X + 0.0429	0.999	8.7 ± 0.3	86.7 ± 3.4	3.9
**8**	UTAS#88	5.10	Y = 0.4251*X + 0.0593	1.000	9.7 ± 0.5	96.6 ± 4.7	4.9
**9**	UTAS#89	6.73	Y = 0.1286*X - 0.0725	0.982	11.6 ± 1.1	116.2 ± 4.9	4.2
**10**	UTAS#54	5.14	Y = 0.6036*X - 0.0735	1.000	9.5 ± 0.2	94.9 ± 2.0	2.1
**11**	UTAS#77	4.79	Y = 0.9435*X - 0.0502	1.000	10.5 ± 0.2	105.0 ± 1.9	1.8
**12**	UTAS#91	5.67	Y = 0.2690*X - 0.0253	0.996	10.8 ± 0.2	107.8 ± 2.5	2.3
**13**	UTAS#95	6.94	Y = 0.3125*X - 0.1449	0.998	10.9 ± 0.4	109.1 ± 3.9	3.9
**14**	UTAS#61	3.00	Y = 1.0490*X + 0.0349	1.000	10.9 ± 0.8	109.5 ± 7.9	7.2
**15**	UTAS#43	3.21	Y = 0.5825*X - 0.0804	0.999	9.7 ± 0.8	96.7 ± 7.8	8.7
**16**	UTAS#46	4.52	Y = 0.9530*X + 0.0376	0.985	11.1 ± 1.1	111.1 ± 10.8	9.7
**Idebenone**		9.05	Y = 0.8976*X - 0.0881	1.000	9.9 ± 1.1	99.1 ± 10.5	10.6

Standards were prepared at 10 μM in 25% ACN and 20 μl was injected. Other conditions are described in Section 2.2, 2.3 and main text. Linear regression of peak area (A) and concentration of standards were generated using GraphPad Prism 8.2.1 with coefficient of determination (R2) calculated. LOQ = 1 μM. 1 mL cell culture media containing 40 μM compounds at ***t*** = 0 were 1:1 precipitated with ACN, vortexed and centrifuged. 1 mL supernatant was 1:1 diluted with purified water, filtered, degassed prior to immidiate RP-LC analysis. Recovered% was calculated by dividing the concentration found at ***t*** = 0 by 10 μM × 100%. Data was expressed as mean ± standard deviation (SD) (n ≥ 3). The repeatability (RSD%) was calculated by dividing the absolute SD by the mean.

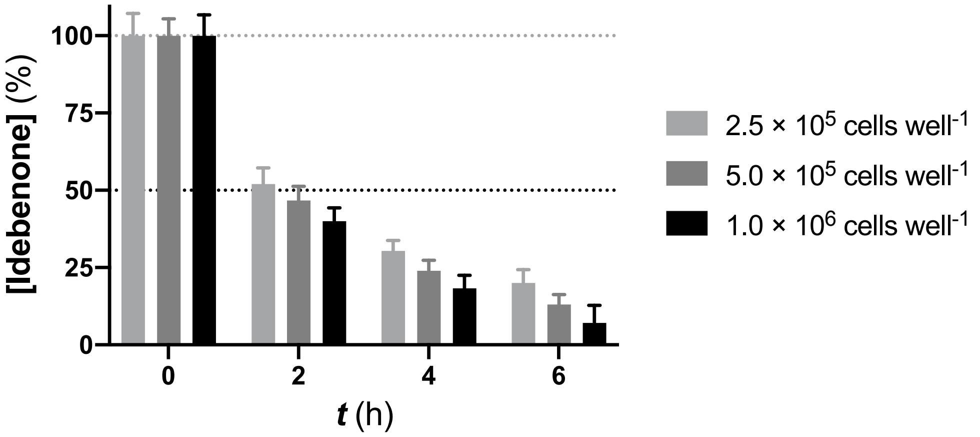

Figure S1. Metabolic conversion of the reference SCQ idebenone over 6 h by different cell densities. Data was expressed as mean ± SD from one experiment, with 3 data points each.

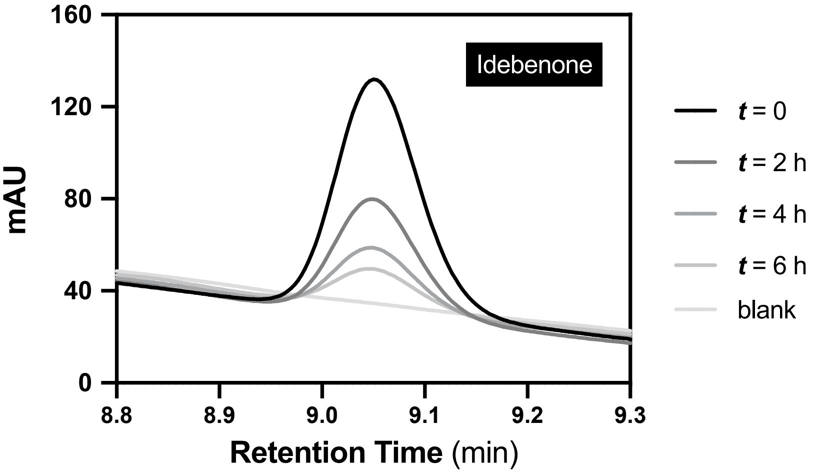

Figure S2. Exemplary chromatograms of SCQ peaks detected after 2, 4 or 6 h metabolism.

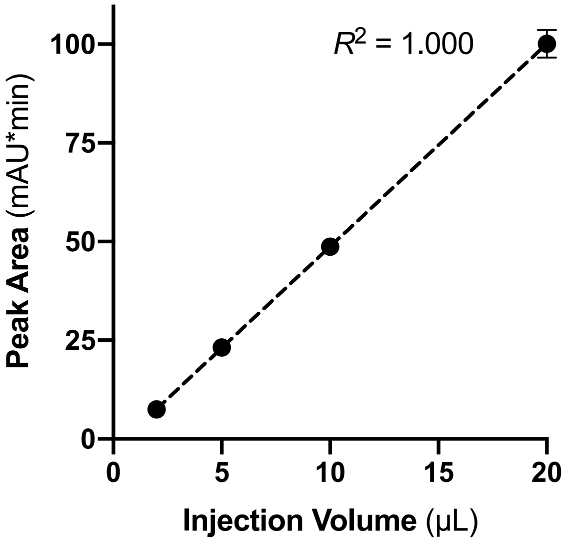

Figure S3. Linear responses of idebenone to injection volumes between 2-20 µL. Data was expressed as mean ± SD from one experiment, with 3 data points each.

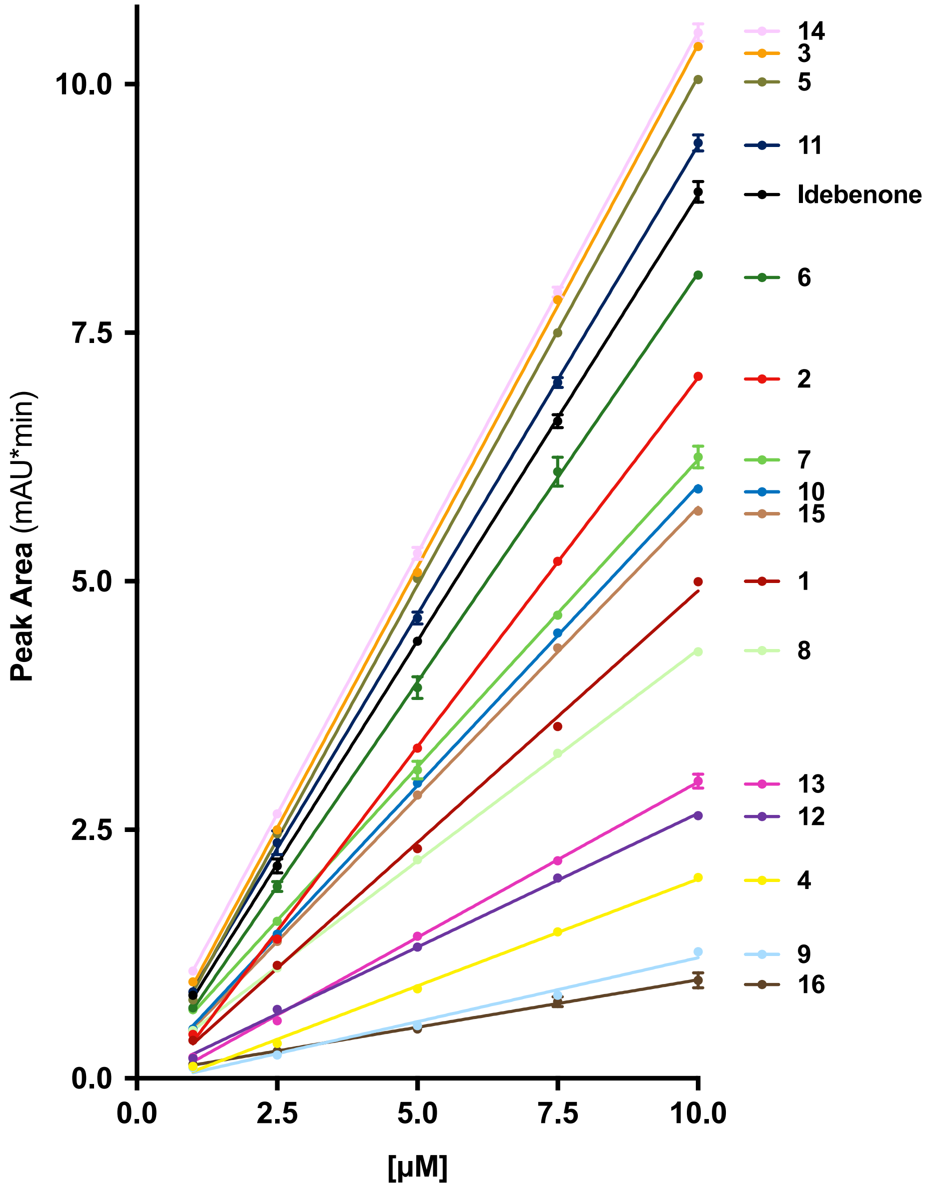

Figure S4. Linear responses of 16 new SCQs and the reference benzoquinone idebenone between 1-10 µM. Data was expressed as mean ± SD from three independent experiments, with 3 data points each.

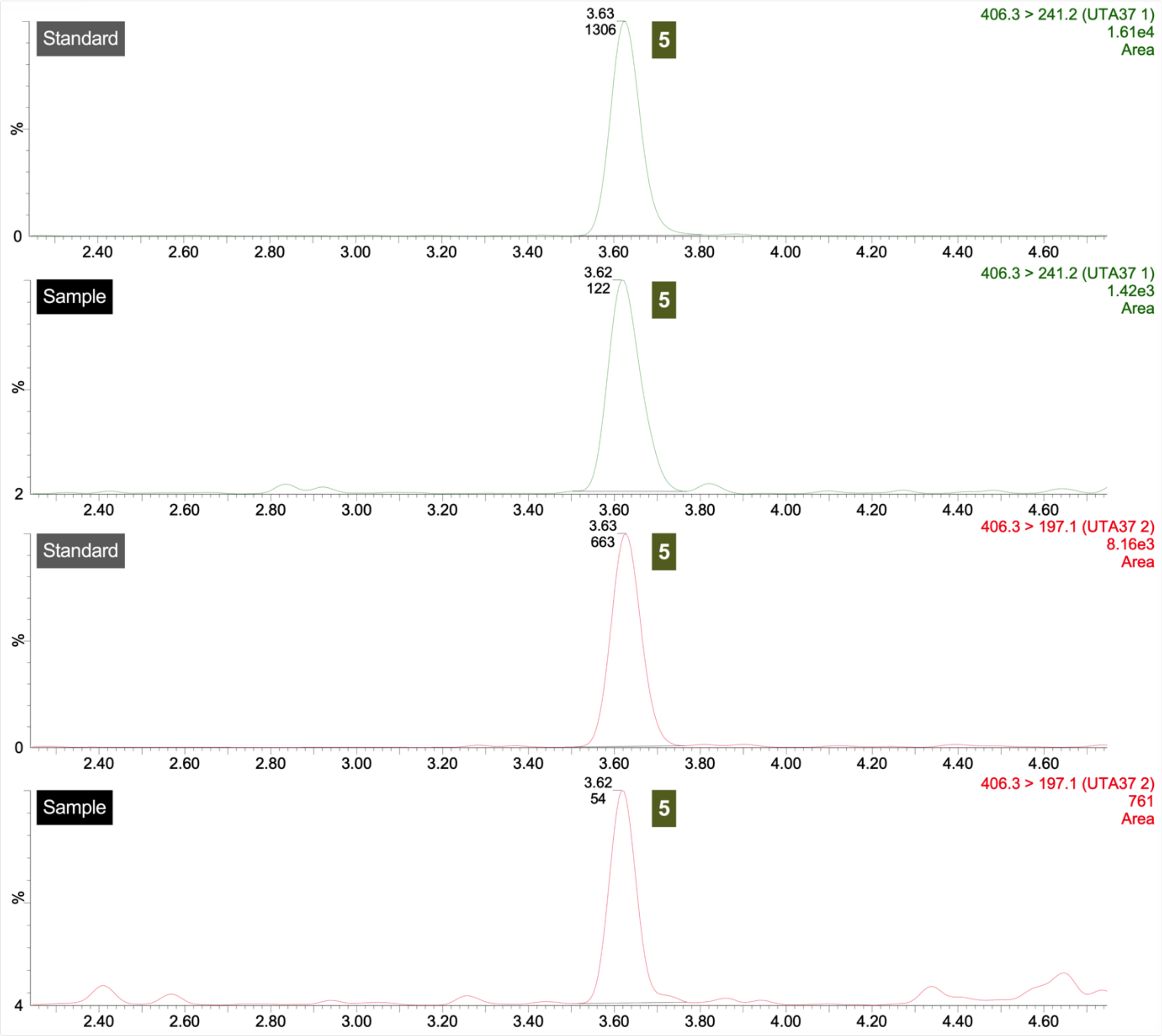

Figure S5. Exemplary mass spectrometry chromatograms for the metabolic conversion from the *L*-phenylalaninol derivative **3** to the *L*-phenylalanine derivative **5**. The detection and quantitation of **5** were performed using a H-Class UPLC-MS/MS system coupled to a XEVO TQ triple quadrupole mass spectrometer (Waters, NSW, AU). Analytical separation was carried out on a Waters Acquity UPLC BEH C18 column (2.1 × 100 mm, particle size 1.7 µm) at 30 °C. Mobile phases were 0.1% formic acid in purified water (A) and acetonitrile (B) with a flow rate of 0.3 mL min^{-1}. The final conditions included a gradient flow of mobile phase B: 40 % for 1 min, 40-90 % for 4 min, 90 % for 1 min, 90-40 % for 0.5 min, 40 % for 3 min (total run t = 9.5 min, including column post-conditioning). The mass spectrometer was operated in positive ionization mode, using electrospray ionization source (ESI). The tuning parameters of **5** were optimized by using a standard solution containing 8.11 ng mL^{-1} **5** (20 nM in acetonitrile) with a flow rate of 20 µL min^{-1} to the mass spectrometer. Cell culture media collected (containing 40 µM **3**) was precipitated by mixing 1:1 with acetonitrile, followed by 1:1000 dilution of the supernatant in acetonitrile to reach a theoretical concentration of 7.83 ng mL^{-1} **3** (20 nM) for analysis. The standards and metabolized samples were detected by monitoring the precursor to product ion transition using Multiple Reaction Monitoring (MRM) scan mode with 78 ms dwell time for each transition. The selected transitions were *m*/*z* 406.3 > 197.1 and 406.3 > 241.2 for **5**. The source temperature was 130 °C, desolvation temperature was 450 °C, desolvation nitrogen gas flow was 950 L h^{-1} and cone gas flow was 50 L h^{-1}. The capillary voltage was set at 2.85 kV, while the cone voltage values for **5** were optimized at 27 V. The multiplier was set at 528 V and argon was used as collision gas. The optimized collision energies were 24 eV (392.2 > 152.1) and 16 eV (392.2 > 197.1), respectively. All data were required using MassLynx software (version 4.0, Waters, NSW, AU).

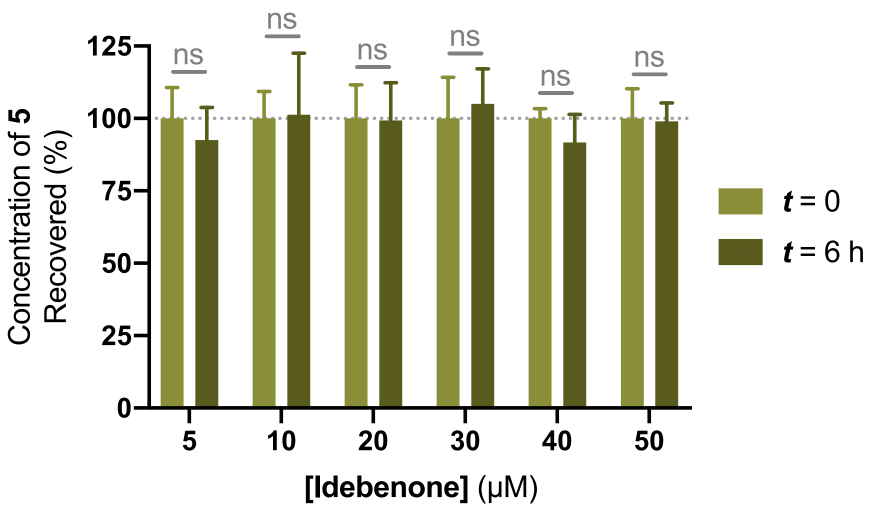

Figure S5. Superior metabolic stability of the *L*-phenylalanine derivative **5** over 6 h in combination with all concentration series of the reference SCQ idebenone. Data was expressed as mean ± SD from three independent experiments, with 4 data points each.

© 2020 by the authors. Submitted for possible open access publication under the terms and conditions of the Creative Commons Attribution (CC BY) license (http://creativecommons.org/licenses/by/4.0/).
